# Development and Validation of the Adolescent and Children Risk of Abuse and Maltreatment Unaccompanied Scale (ACRAM-US)

**DOI:** 10.1007/s40653-024-00627-4

**Published:** 2024-03-09

**Authors:** Paula Samper, Adrián García-Mollá, José M. Tomás, Elisabet Marco-Arocas

**Affiliations:** 1 Department of Basic Psychology, University of Valencia, Valencia, Spain; 2 Department of Methodology for the Behavioral Sciences, University of Valencia, Blasco Ibáñez Avenue, 21, Valencia, 46010 Spain; 3 Department of Sociology and Social Anthropology, University of Valencia, Valencia, Spain

**Keywords:** Child Abuse and Neglect, Unaccompanied asylum-seeking children and adolescents, Migration, Assessment, Validity

## Abstract

Independent migration of children and adolescents is becoming a political and social issue in recent years. Literature documents that the migration process of young people without an adult referent entail serious psychological problems. Moreover, the lack of coherence in the assessment and care processes aggravates the damage suffered by minors, which requires a greater investment of institutional resources. The aim of this research is to describe the development and provide psychometric properties of Adolescent and Children Risk of Abuse and Maltreatment Unaccompanied Scale (ACRAM-US), a 9-items scale for the assessment of risks factors of child abuse and neglect in the specific population of unaccompanied asylum-seeking children and adolescents. Structural validity, reliability and convergent-related validity were studied for this measure in a sample of 128 unaccompanied children and adolescents. The sample included cases of 14 different nationalities. Children’s mean age was 16.94 (SD = 1.84), and 96.9% were male and 3.1% were female. Cases were informed by child welfare workers from different protective services in the XXXX Community (XXXX). The results of Exploratory Factor Analyses (EFA) indicate performance better solution with 2-dimensions which was also in line with theoretical formulation (*χ*^2^ = 31.55, *df* = 19, *p* = .035, CFI = 0.991, SRMR = 0.081, RMSEA = 0.072, [90% CI: 0.019 − 0.115]). Results of convergent validity showed significant correlation with the Children Trauma Questionnaire-Short Form (CTQ-SF). Therefore, this study provides data of the first scale that assess risks factors of maltreatment for the unaccompanied asylum-seeking children and adolescents.

## Introduction

Migration in childhood has traditionally been understood as part of a family strategy, where children and adolescents have been described as passive victims of adult decisions and may be subjected to exploitation and conflict. Consequently, the active role in decision-making in the migration process has been made invisible (Hashim, 2016). Many studies on children’s mobility have questioned how childhood has been constructed historically and reveal the complexity and diversity of experiences they live in the migration process (Gardner, 2012; Hashim & Thorsen, 2011; Jiménez, 2011; Jiménez & Trujillo, 2019; Suárez, 2006; Whitehead & Hashim, 2005). Thus, migrant childhood encompasses a set of diverse phenomena: children who are part of a migrant family, who live a transnational affiliation, who are born in the destination countries, who undertake migratory projects independently or who return to their countries of origin, among other (Pavez-Soto, 2017). Specifically, the term independently migrating children is used to refer generally to children who migrate without the company of their primary caregivers, although the decision may or may not be autonomous (Hashim, 2006).

This paper focuses on children and adolescents who migrate independently, known in legal terms in Spain as “unaccompanied foreign minors”. In 1997, the Resolution of the Council of the European Union introduced this term to define individuals under 18 years of age who arrive in territory of the member states without being effectively taken into the care of an adult responsible for them, either legally or in accordance with custom and practice. Subsequently, Directive 2001/55/EC completed the definition by mentioning minors who are left unaccompanied after their arrival on the territory of the member states (Arce, 2020). Spain, together with France and Italy, are examples of European states that allow for the reception and protection of unaccompanied minors on the basis of their status as minors deprived of responsible adult custodians. In other states in the European context, that protection is conditional on a prior request for asylum and, therefore, they are defined as “unaccompanied asylum-seeking foreign minors” (Senovilla-Hernández, 2017).

The number of unaccompanied asylum-seeking or ‘separated’ children moving across borders on a global scale has increased drastically in recent decades (García & Birman, 2022). This is observed in the growing scientific literature from different countries such as Germany (Aflaki & Freise, 2019), Spain (Bravo & Santos-González, 2017), USA (Chávez & Menjívar, 2010), Ghana (Hashim, 2005), UK (Thomas et al., 2004), or Sweden (Thommessen et al., 2015), just to reference a few examples. As stated by the United Nations High Commissioner for Refugees (2020), there is an increasing incidence of these cases in different regions of Central America, North America and Europe. This report also points out at different ways of violence, abuse, abandonment and exploitation to which unaccompanied migrant children are subjected and, therefore, the establishment of appropriate international protection measures.

The situations, objectives and expectations that motivate the irruption of these children and adolescents into the field of migration on a global scale are multiple. These are complex decisions where they take great risks in dangerous ways of travelling and circumventing borders that may entail serious consequences. As this is a very difficult decision with serious consequences for the individual, the reasons behind this flow of migration are usually situations of violence, poverty or war in the country of origin (Hopkins & Hill, 2010). As argued by Jiménez (2015), the mobilization of children and adolescents in the global migration context is related to the rupture of dependency systems, selective border processes and the perception of dependency as a resource that allows for movement in a transnational context.

This particular population is highly vulnerable to a wide range of risk situations for the inherent nature of the phenomenon. In most cases, the only way to cross the border is through organized gangs that try to make a financial profit from this process, which means that young people can be abused by smugglers (Sawyer & Márquez, 2017). It also requires special attention the situation of children brought by other adults who have no intention of caring for them, human trafficking for the purpose of sexual or other exploitation, one of the rapidly increasing areas of international criminal activity (Bump & Duncan, 2003). Once in the country of destination, young people face the possibility of deportation or imprisonment. This is mainly due to the double logic of the legal term “unaccompanied foreign minor”: they are minors to be protected but also foreigners to be controlled. There is a confrontation between two forms of governance: that of child protection, which obliges States to recognize and extend the rights as conveyed in the Convention on the Rights of the Child (CRC), and that of migration control, which curtails these rights in different ways (Arce, 2016; Jiménez, 2019). The constant interplay between protection and control, which is not always resolved in the best interests of children and adolescents, is at the heart of the failure of states to respond to the needs of children and adolescents (Hek et al., 2012). In the case of countries where it is necessary to apply for asylum, they face great difficulties in obtaining the appropriate documentation, an important issue that generates a scenario of legal and administrative invisibility and lack of protection for those who do not meet the requirements or do not wish to apply for asylum (Senovilla-Hernández, 2017). Finally, it should be noted that the vision of the problem of the mobility of these young people and the logic of control that underlies institutional and administrative practices, the reception and the resources provided to care for them, exposes them to situations of helplessness and extreme vulnerability.

In the case of Spain, France and Italy, the different forms of institutional mistreatment (age determination test as a filter for access to protection, repatriations, lack of administrative guardianship, denial of documentation…) generate the flight of these children and adolescents from the Spanish protection system and their mobility to other European Union countries, as is the case in Sweden (Montesinos & Jiménez, 2015).

In Spain, the presence of children and adolescents who migrate alone began to become visible in the late 1990s. In the early 2000s, it became more widespread as it became part of the regional protection systems and progressively became a structural phenomenon (Quiroga & Chagas, 2021).

Throughout these two decades in Spain, it has been difficult to precisely quantify the volume of unaccompanied foreign minors in the protection systems. In its annual reports, Defensor del Pueblo (2020) has highlighted the difficulty in obtaining reliable data, due to the large fluctuations in the figures and the significant differences between the data provided by the regional child protection services and those provided by the Register of Unaccompanied Foreign Minors at the State level. These differences are caused by several factors, among them the high mobility of children between the different autonomous communities, without a reliable reflection of registrations and deregistrations. While in the 2020 annual report issued by Defensor del Pueblo there were 9,030 minors registered in the register (869 were girls), the Ministerio de Derechos Sociales y Agenda 2030 (2020) reports a total of 5670 cases, of which 5579 in residential care and 91 in foster care.

As the data show for the Spanish territory, this is a problem that mainly concerns boys. The number of girls is far from the size and significance of male migration in the protection system, which does not mean that there are no girls involved in this process (Lázaro, 2007). On the contrary, their low visibility in protection systems reveals the intense vulnerability to which girls are exposed. In addition to the risks involved in the migration of minors, there are factors of vulnerability and precariousness derived from their gender (Diaz, 2017). Many of these girls are victims of sexual and/or labor exploitation, in a context where it is difficult, if not impossible, for them to access the protection system (Morante & Trujillo, 2014). Unaccompanied foreign minors arriving in Spain come from various countries, mainly from the Maghreb and West African areas, with Morocco being the country of origin of most of them throughout the history of the phenomenon (Jiménez, 2019).

Specifically, in the Valencian Community, the presence of these migrant minors in the regional protection system has increased since 2004, becoming over the years one of the groups most present in the regional protection system (Horcas, 2016; Marco, 2017). While in the early years it seemed to be a transit territory towards other destinations, it ended up becoming a destination territory for many of these young people. This is related both to the difficulties in settling in neighboring communities and to the network of resources available to care for them in this territory (Marco et al., 2020).

In the case of the Valencian Community, the structure of residential foster care resources distinguishes between Homes and Residences depending on the number of persons. In addition, these are differentiated according to their characteristics: reception, specific for serious behavioral problems and general foster care. Reception residences are intended for immediate care or first reception, being the gateway to the network of resources. One of the main objectives of these resources is to prepare an interdisciplinary diagnosis of the personal, social and family situation of the children and adolescents in care and their environment in order to propose the protection measure and referral to the most appropriate resource. The period of stay in these residences will be limited to the time strictly necessary to carry out these study functions, being the maximum period of days from the assumption of provisional guardianship, extendable to another 45 days when circumstances so require, as is specified in Ley 26/2018 (2019).

As explained by da Silva Rebelo et al. (2018), society’s discriminatory attitudes may result in the avoidance of social and health services by refugees and asylum seekers producing a harmful impact on their biopsychosocial well-being. As explained in the study carried out by (Oppedal & Idsoe, 2015), the situation of unaccompanied foreign minors requires an interdisciplinary intervention by psychotherapists, social workers and teachers. This is because there are large cultural differences (both with locals and workers) that must be mitigated through a long and arduous process of cultural adaptation Wernesjö (2012). With regard to the psychological consequences of this migratory process, there is a large body of research that has determined that this is a situation that predisposes to the development of several mental health problems like post-traumatic stress disorder or major depressive disorder (Behrendt et al., 2022; Cardoso, 2018; Ehntholt et al., 2018; Schwartz et al., 2015). A relevant point in the approach of psychological interventions is the assessment and development of necessary adaptations for this population. They must be carried out taking into account their ethnicity, language and literacy, requiring the intervention of interpreters for the achievement of objectives (King & Said, 2019).

In this line, a marked difference has been found between individuals with higher or lower symptoms depending on their psychosocial functioning, family relationships or cultural belonginess (McGregor et al., 2015). In relation to the psychosocial outcomes, McEwen et al. (2022) showed that unaccompanied young migrants present more externalizing than internalizing symptoms. Finally, as reported by Mittendorfer-Rutz et al. (2020), the mental health consequences for unaccompanied foreign minors can be fatal given the high suicide rate among this population. Despite the difficulties and adverse situation involved in migrating without an adult reference, unaccompanied asylum-seeking children have demonstrated to be resilient (Jafari et al., 2022; Pieloch et al., 2016) and able to generate social support networks that serve as a protective factor against psychosocial problems (Keles & Oppedal, 2022).

Reviewing the scientific literature, we could find a lot of qualitative studies regarding the nature of the phenomenon and the exposition to risk situations of migrant youths using interviews and testimonies (Garcia & Birman, 2022; Thomas et al., 2004; Thommessen et al., 2015), but quantitative studies are scarce. This is in addition to the need for migrant children to be recognized as refugees in order to have access to basic services and to receive the institutional protection they require from organized violence and war situations. Asylum recognition is subject to a rigorous assessment that is sometimes detrimental to the welfare of children (Given-Wilson et al., 2016). For this reason, it is necessary to dispose of tools to recognize institutional abuses and to provide a basis for interventions tailored to the needs of unaccompanied foreign minors. In this line, there is a need to implement comprehensive assessment tools with good psychometric properties that allow the recognition of risk situations related to structural and cumulative factors (Lamela & Figueiredo, 2018; Yang & Maguire-Jack, 2018).

## Current Study

ACRAM (Adolescent and Children Risk of Abuse and Maltreatment) arises as a response to the need and demand of professionals, experts, and researchers (WHO, 2020b; Unicef, 1989) to have a common, valid and reliable tool that makes it possible to draw up standardized and objective diagnoses, to guide professional decision-making, and to assess situations of risk and lack of protection of children and adolescents. Additionally, having a scale available may lead to more quantitative research in this area. ACRAM is a battery of questionnaires covering parental and caregiver risk factors (ACRAM-PS), community-related factors (ACRAM-CS), protective factors (ACRAM-PFS) and, of relevance here, other factors related to the complexities associated with unaccompanied asylum seeker children (ACRAM-US). For the best of our acknowledgment, there’s a lack of studies in the literature dealing with the risk of maltreatment and its measurement on unaccompanied asylum-seeking children. Given the absence of specific tools to assess abuse and neglect on this particular population, the general aim of this study was to develop and provide an actuarial risk inventory for the adequate assistance of children and adolescents involved in this situation. Moreover, this study, specifically, intended to develop and validate the ACRAM-US scale, including the analyses of factor structure, reliability and convergent validity.

## Methods

### Sample and Procedure

The current study is part of the ACRAM project, a longitudinal research that aimed to develop and validate a battery of scales of determinants on child maltreatment in the Valencian Community (Spain). A convenience sample of unaccompanied migrant children was collected for this study. For this purpose, centres assisting specifically unaccompanied asylum-seeking children were located and selected, and professionals working on them were proposed to participate in the study. They provided data about the cases they were attending to or had attended to recently using an online survey.

Finally, data was gathered for 128 cases of children and adolescents assisted by different child welfare workers. From this total sample, 96.9% were male and 3.1% were female. Their mean age was 16.94 years (*SD* = 1.84). The sample comprised 14 different nationalities, the most represented were from Maghreb countries including a 76.6% of cases from Morocco and a 3.9% from Algeria. Other African nationalities represented major part of the rest of the sample: Ghana (3.1%), Ivory Coast (2.3%), Gambia (2.3%), Mali (2.3%) and Senegal (2.3%). A total of 21.1% of the cases have been living in Spain for more than 3 years, 46.9% between 1 and 3 years and 32% less than 1 year.

This research complied with APA’s ethical standards, and it was approved by the Ethical Commission of the Valencian Government (CSV:HYH5NVSA-Y85ZSB11-RML6ZCYX). The children’s data were all anonymous, and all professionals signed informed consents.

### Instruments

Two scales were employed in this research: the one we developed and validated (ACRAM-US), and the Childhood Trauma Questionnaire—Short Form with validation purposes.

The ACRAM (Carbonell et al., 2023; Navarro-Pérez et al., 2023) is a battery of scales for the detection and assessment of child maltreatment including 97 indicators grouped into three different scales: (1) Risk factors associated to parental/caregiver behaviour (2) Risk factors associated to the environment, and (3) Protective factors. Additionally, a fourth scale for complementary use was also developed for the specific case of unaccompanied asylum-seeking children. In this study, the latter scale called Adolescents and Children Risk of Abuse and Maltreatment Unaccompanied Scale (ACRAM-US) is examined. ACRAM-US is a risk indicators inventory designed to be filled in by child welfare professionals. During the theoretical development of the scale, it was pretended to include indicators related to these children or adolescents’ inherent risks and the situations of institutional abuse. The scale had 9 items or indicators rated on a three-point Likert scale: 0 (there is clear evidence it does not occur), 1 (there are signs it might occur, but it cannot be confirmed) and 2 (there is clear evidence it does occur). Thus, the greater the number of risk indicators found, the greater the risk of maltreatment.

The Childhood Trauma Questionnaire-Short Form (Bernstein et al., 2003). The CTQ-SF is a 28-item self-reported measure of retrospective child abuse and neglect, this short version was developed from an initial version comprised of 70 items (Bernstein et al., 1994). This study employed a Spanish adaptation of CTQ-SF designed to be filled out by child welfare workers. This scale comprises five different types of maltreatment: emotional abuse (EA), physical abuse (PA), sexual abuse (SA), emotional neglect (EN), and physical neglect (PN). The scale presented adequate reliability for this sample with α = 0.89 for EA, α = 0.95 for PA, α = 0.96 for SA, α = 0.86 for PN and α = 0.97 for EN. Twenty-five items measure all of scales scoring on a five-point Likert scale from 1 (*never true*) to 5 (*very often true)*. Moreover, the scale includes three items to discern negative childhood experiences or socially desirable responses.

### Statistical Analyses

Exploratory Factor Analyses (EFAs) were performed in order to assess the factorial structure of the ACRAM-US. These EFAs estimated from one to three factors. Given the theoretical design of the questionnaire, the best fitting solution was expected to be the two-factor model. The estimation method employed on this study was WLSMV, because performs the best when categorical non-normal data have to be analysed (Finney & DiStefano, 2013). Fit was addressed with several statistics and indexes, specifically: the chi-square statistic (χ^2^), the Comparative Fit Index (CFI), the Standardized Root Mean Square Residual (SRMR), and the Root Mean Squared Error of Approximation (RMSEA). Acceptable model fit is considered in the presence of CFI values equal or greater than 0.90 and RMSEA values equal or lower than 0.08 according to Hu and Bentler (1999). Analyses were performed using Mplus 8.7. (Muthén & Muthén, 1998–2017). Descriptive statistics and correlation coefficients were calculated in SPSS 26. Correlations were also calculated between the ACRAM-US and CTQ-SF to test for convergent validity. Finally, internal consistency of the ACRAM-US dimensions was estimated using McDonald’s Omega (McDonald, 2013) in order to overcome limitations of Cronbach’s alpha (Hancock & An, 2018).

## Results

### Factor Structure

Given de absence of an a priori hypothesis concerning the factor structure, EFAs were run, estimating one to three-factors, with all 9 items of the initial version of the ACRAM-US. These three-factors structures can then be compared through their relative fit to the data. Fit indexes for the three solutions and models comparison can be consulted in Table 1. In general, best fit indexes were those of the two factors model: *χ*^2^ = 31.55, *df* = 19, *p* = .035, CFI = 0.991, SRMR = 0.081, RMSEA = 0.072, [90% CI: 0.019 − 0.115]. Geomin rotated loadings for the two factors model are presented in Table 2. All standardized factor loadings were statistically significant (*p* < .01) and large.

**Table 1:** Goodness-of-fit indices for the tested models

Models	χ^2^	df	p	RMSEA	90% CI	SRMR	CFI
1. One-factor	61.905	27	< 0.001	0.100	0.068-0.134	0.145	0.974
2. Two factors	31.550	19	0.035	0.072	0.019-0.115	0.081	0.991
3. Three factors	19.516	12	0.076	0.070	0.000-0.124	0.054	0.994
4. One-factor vs. two factors	28.028	8	< 0.001				
5. Two factors vs. three factors	12.385	7	0.088				

**Table 2:** Unstandardized factor loadings, correlation coefficients among factors and omega coefficient for each factor

		Inherent risks to unaccompanied minors (F1)		Institutional maltreatment to unaccompanied minors (F2)
Item 1			0.679*	
Item 2			0.474*	
Item 3				0.796*
Item 4				0.930*
Item 5			0.742*	
Item 6			0.942*	
Item 7			0.930*	
Item 8			0.814*	
Item 9			0.794*	
Factor correlations	*F1*		1	
	*F*2		0.35*	1
Omega coefficients		0.940		0.849

*Note*: * = *p* < .05

### Internal Consistency

The scale has shown adequate internal consistency in the sample. McDonald’s omega for each factor were ω = 0.940 for inherent risks to unaccompanied asylum-seeking children and ω = 0.849 for institutional maltreatment to unaccompanied children. Omega coefficients are presented in Table 2.

### Convergent-Related Validity

Correlations among the ACRAM-US scores and the CTQ-SF are presented on Table 3. Inherent risks to unaccompanied asylum-seeking children dimension showed significant and positive correlations with physical abuse and physical neglect factors from CTQ-SF, while institutional maltreatment to unaccompanied asylum-seeking children presented significant and positive correlations with emotional abuse and physical neglect. As expected, correlations between dimensions comprised on the ACRAM-US were statistically significant and positive. Correlation coefficients among the ACRAM-US dimensions and the CTQ-Sf dimensions are included in Table 2. Overall, we can conclude that the questionnaire has adequate convergent-related evidence of validity.

**Table 3:** Correlation coefficients among measures’ dimensions

Measures’ dimensions		CTQ-SF				
		EA	PA	SA	EN	PN
Unaccompanied Minors Risk Factors Scale	IR	− 0.06	0.32**	0.18	0.04	0.23**
	IM	0.26**	0.01	0.01	0.12	0.36**

*Note*: ** = *p* < .01; * = *p* < .05

Unaccompanied Minors Risk Factors Scale: inherent risks to unaccompanied minors (IR) and institutional maltreatment to unaccompanied minors (IM).

CTQ-SF: emotional abuse (EA), physical abuse (PA), sexual abuse (SA), emotional neglect (EN), and physical neglect (PN).

## Discussion

The current study aimed to develop a scale to measure risks factors in unaccompanied asylum-seeking youth and also provide evidence of the psychometric properties of this measure. This scale is integrated into a battery of risk and protective indicators of child maltreatment as a complement to the specific assessment of the situations to which unaccompanied asylum-seeking youth are exposed. Given the increasing migratory flow of this population, this assessment tool allows for a better assessment, and therefore an improvement in the care provided to these young people, in line with the conclusion drawn by Bravo and Santos-González (2017) and Newbigging and Thomas (2011). For this reason, the use of the complete ACRAM battery in this population allows the achievement of the objectives of promoting positives traits set out by the research carried out by Ní Raghallaigh and Gilligan (2010). In addition, the ACRAM-US also responds to the need to have available, comprehensive, unbiased and psychometrically sound assessment tools (Brumley et al., 2019; Gabrielli & Jackson, 2019; Kugler et al., 2019). The scale was designed for its use in Spanish speaking countries. In particular, this study attempts to provide evidence on the structural validity, internal consistency and convergent validity of the scale.

In this line and regarding the first specific aim, we explored the factor structure in the total sample using EFAs from 1 to 3 factors. Model fit indices showed a very good fit of the structure to the data and all items loaded significantly on their corresponding dimensions. As it was expected, the two-factor solution was the one with the best fit indexes given the construction of the scale based on factors related to the nature of the unaccompanied asylum-seeking youths (Garcia & Birman, 2022) and the institutional and system maltreatment they receive for the only reason to be migrant (Given-Wilson et al., 2016). This responds to the need to use a model that integrates information from different systems to allow practitioners and researchers to make more accurate and comprehensive assessments for the child maltreatment (Begle et al., 2010).

We estimated internal consistency using McDonald’s omega based on standardized factor loadings from the best fitting model. The results suggested that ACRAM-US displays an adequate internal consistency with all omegas been more than 0.70.

In respect to criterion-related validity, the ACRAM-US dimensions are all correlated in the expected direction. In general, the ACRAM-US dimensions presented significant positive correlation coefficients with CTQ-SF (Bernstein et al., 2003). Regarding the CTQ-SF, dimensions of physical abuse and physical neglect, are better related with the inherent risks of unaccompanied minors dimension of ACRAM-US. Furthermore, the institutional maltreatment dimension of ACRAM-US is better related to emotional abuse and physical neglect.

The results of this study show that ACRAM-US has the optimal psychometric properties to assess risk factors of maltreatment on the specific population of unaccompanied young migrants. This scale, used integrated in the ACRAM battery of scales, covers the need to assess the complex reality of child maltreatment given the requirement of comprehensive assessments that take into account both negative and positive aspects (Calheiros et al., 2021). Furthermore, given the pandemic caused by COVID-19, the attention and protection of children is particularly important, as they are known to be a particularly vulnerable population in the face of such disasters (Galea et al., 2005). Particularly in a context where social isolation and economic stress resulting from the pandemic may have exacerbated the risk of abuse (Self-Brown et al., 2022; Lee et al., 2021). Research on children’s well-being in adverse situations emphasizes the importance of individual, family, and environmental resources in promoting positive development and outcomes in the face of disasters (Zhang et al., 2020).

This study shows up the relevance of an adequate attendance to the unaccompanied asylum seeker children given the intrinsically vulnerability of this population and the greater exposure to adverse situations and institutional maltreatment (Given-Wilson et al., 2016). As exposed by Arredondo et al. (2017), the governmental and non-governmental institutions have an obligation and a responsibility to provide optimal care to ensure the well-being of the child by providing resources and access to basic services. As aforementioned, the use of the ACRAM battery (including the ACRAM-US) is an advantage for the child welfare professionals attending unaccompanied asylum-seeking youths to ensure their well-being after a migration process that generates harmful consequences on their development (Ehntholt et al., 2018). Finally, the implementation of the ACRAM scale also represents a significant progress in the assessment of cases by welfare professionals since the development of software (DAPware) has been carried out to facilitate reporting and evaluation over time (Navarro-Pérez et al., 2023).

### Limitations

This study also present limitations. The first one refers to the lack of information coming from the actors of this migratory phenomenon about the processes of arrival in a new country and the bureaucratic processes. This scale was developed based on the information offered by the child welfare professionals who assess and attend these cases. Another limitation of this study is that the sample was relatively small and gathered by a non-probabilistic method, which means that conclusions may not be extrapolated to other settings were political and welfare processes variate, and also that cannot be considered representative of the population. Finally, and in this line, the results of this study are difficult to generalise internationally given the great differences in the assessment and care processes for migrant children and the variation in the factors that motivate their migration according to the country of origin and destination.

As far as we know, this is the first study on the development and validation of an objective and rigorous measurement scale on risk indicators in unaccompanied asylum seeker youth. Given the increasing migratory flow of this type of population, this assessment tool allows for an improvement in the care provided to these young people, in line with the conclusion drawn by Newbigging and Thomas (2011). The ACRAM-US is integrated into a battery of risk and protective indicators of child maltreatment as a complement to the specific assessment of the situations to which unaccompanied asylum-seeking youth are exposed. For this reason, the use of the complete ACRAM battery in this population allows the achievement of the objectives of promoting positives traits set out by the research carried out by Ní Raghallaigh and Gilligan (2010). In contrast, although we have developed the scale with the help of the workers of the protection systems, it would be interesting to have information on the processes of arrival in a new country and the bureaucratic processes from the perspective of the actors of this migratory phenomenon. Another limitation of this study is that sample was reduced and gathered by a non-probabilistic method, which means that conclusions may not be extrapolated to other countries were political and welfare processes variate. Finally, and in this line, the results of this study are difficult to generalise internationally given the great differences in the assessment and care processes for migrant children and the variation in the factors that motivate their migration according to the country of origin and destination.

## Conclusions

Given the vulnerability presented by unaccompanied asylum-seeking youths and increasing incidence rates in the recent years, emerges the need to the creation and implementation of a risks assessment scale specifically designed for the risk situations involving this population. Current study aimed to develop and provide evidence of the adequate psychometric properties of the ACRAM-US, a specific scale designed to assess risk situations on unaccompanied asylum-seeking children. It is included in the ACRAM battery which is a comprehensive instrument for the detection and assessment of child maltreatment. The ACRAM-US constitute the first scale on the assessment of specific risks of this population and must be used with the rest of scales comprised on the ACRAM for the exhaustively evaluation. The results support that ACRAM-US is a reliable and rigorous measure for assessing risks factors of unaccompanied youths and can serve as a key tool for designing new intervention strategies.
